# Do the Indonesians Receive the Dental Care Treatment They Need? A Secondary Analysis on Self-Perceived Dental Care Need

**DOI:** 10.5402/2012/769809

**Published:** 2012-07-19

**Authors:** Diah Ayu Maharani

**Affiliations:** Department of Preventive and Public Health Dentistry, Faculty of Dentistry, University of Indonesia, Jl. Raya Salemba No.4, Jakarta 10430, Indonesia

## Abstract

Increasing access for citizens to health services, including dental care, is one of the primary targets of the Indonesian Ministry of Health. To assess progress toward this goal, we sought to describe the magnitude of unmet needs for dental care among Indonesians. Secondary data of nationally representative surveys conducted from 2003 to 2007 were analysed to describe the associations between unmet needs for dental care in different demographic groups. In average, 2.28% of the Indonesian population reported perceiving need for dental care and 0.74% reported utilizing dental care. The average of unmet need was 72.04%. Logistic regression analysis indicated that respondents who lived in rural areas, who were uninsured, had higher odds ratios in reporting unmet dental care needs. Perceived need for and utilization of dental care among Indonesians was found to be low. Moreover, the unmet need for dental care is relatively high.

## 1. Introduction

In a WHO [1] publication, it is stated that one of the primary targets of the Indonesian Ministry of Health is to increase access for citizens to health services, including dental care. But facts indicated that dental health problems can still be found in almost every area in Indonesia. The Indonesian Basic Health Survey 2007 showed that the rates of edentulous were 2% of the whole population, and only 4.5% of them used dentures. The National DMF-T (Decayed Missing Filling Tooth) index was 4,85. The biggest component was missing teeth (M-T), which was 3.86, describing that in average every Indonesian has 4 teeth extracted or was indicated for extraction. Moreover, 17.6% of Indonesians aged 65 or older had lost all their teeth. This percentage is far from the WHO target of less than 5% edentulous for year 2010 [2]. 

One of the oral health objectives of the Indonesian Ministry of Health for 2010 is to increase in the proportion of Indonesians who utilize dental health care annually [3]. Given this situation, it is important to evaluate the extent of those who have a need for dental care but do not receive any dental treatments or in other words those who had unmet dental care needs. This kind of evaluation is important to study the impact of government's policy to dental health in Indonesia. Nevertheless, there is a lack of information about how dental health care have changed over time causing the inability to assess the effects of government policies, whether these policies were leading toward or away from greater social justice. Therefore, the objective of this study was to assess the magnitude of unmet need for dental care in the Indonesian population.

## 2. Materials and Methods

We undertook evaluations of unmet dental care need using secondary datasets of the Indonesian population from the Indonesian National Socio Economic Survey (Susenas) interviews data conducted between 2003 and 2007. Data on self-reported dental care need experienced in the month preceding the survey might not adequately estimate the real need for dental care. This might be due to different subjective perception and expectation regarding health across socio-economic groups. Inspite of this, previous study had demonstrated that self-assessment indicators have been effective in capturing health variation in a population [4].

Susenas is an annual, continuous, multipurpose, cross-sectional, nationally representative survey of the Indonesian population conducted by the Indonesian National Board of Statistics. It uses cluster sampling, sorted geographically by province, and it includes demographics and also data on perceived need for and utilization of dental care. The total numbers of respondents in 2003 were 895,427 subjects, 2004 were 1,030,250 subjects, 2005 were 1,052,091 subjects, 2006 were 1,107,594 subjects, and in 2007 were 1,167,019 subjects. This included individuals of all ages across all of the provinces in Indonesia. The data were weighted to ensure that the sample was representative of the Indonesian population. 

Respondents were asked about their self-perceived need for dental care in the preceding month and whether they obtained care for that need. This information was obtained by means of a single question in the interview. The responses were categorized as either yes or no responses. The unmet need, that is, those who had perceived need for dental care but did not received dental treatment, was the response variable in this study. A set of explanatory variables that were found in previous studies to be important predictors and that were available in the Susenas data was selected as a set of possible factors affecting perceived need for and utilization of dental care services [5–8]. In the present study, the independent variables were comprised of age (<15, 15–29, 30–44, 45–59, 60< years), gender (female or male), residence (rural or urban), macroregions (Sumatra, Java, Lesser Sunda Islands, Kalimantan, Sulawesi, and Maluku Islands including West Papua), and health insurance entitlement (uninsured or insured). 

Descriptive analyses were performed for all variables. Associations between categorical variables were determined by chi-square test. To examine the association between dependent and independent variables, logistic regression analysis was performed, considering unmet need for dental care (0: no and 1: yes) as a response variable. Explanatory variables were included in the model as covariates by using a procedure for variable selection in which all variables in a block were entered in a single step. The adjusted odds ratios and 95% confidence intervals were calculated. In the statistical analyses, dummy variables were used for categories with 3 or more groups. A significance level of 0.05 was used throughout to denote statistical significance. For statistical processing, SPSS statistical software version 13.0 was used.

## 3. Results

Table 1 shows the distribution of perceived need for and utilization of dental care among respondents of Susenas from 2003 to 2007. The percentage of Indonesians who perceived a need for and utilized dental care from 2003 to 2007 was low. The average number of respondents who reported perceiving need for dental care was 2.28%. The average utilization rate of dental care was 0.74%. As shown in Table 2 among those who reported having a need for dental care, in average, only 32.06% sought dental care treatment. Moreover, we found that, in average, 72.04% of respondents between 2003 and 2007 had unmet needs, those who perceived need for dental care but did not receive any dental treatment. Unmet need for dental care was greater for individuals aged 15–29 and 30–44, those in rural areas, and those who were uninsured (Table 3). Unmet dental care needs were only slightly different between males and females. 

The adjusted odds ratios (ORs) and 95% confidence intervals (CIs) for unmet dental care need that were associated with some explanatory variables (age groups, gender, residence, macroregions, health insurance entitlement) are listed in Table 4. The explanatory variables that were included in the model were significantly associated with unmet need except gender. Overall the uninsured respondents and those who were in the 15–29 year age group yielded strong associations with unmet need for dental care. 

## 4. Discussion

A population's need and utilization of dental services is an important parameter of dental health care planning [9]. Although improvement of the community's dental health is a central goal of dental health care interventions, only little attention has yet been paid to changes in subjective or self-perceptions regarding dental health [10]. This study revealed that despite the national prevalence of caries was 43.4% only 2.28% of the respondents of Susenas perceived a need for dental care. The unmet need for dental care was relatively high. Among those who reported having problems with their dental health, 67.95% did not receive any treatment. The low dental care utilization rate may be due to economic and geographic barriers to access dental care the existing.

In general, the unmet dental care need was higher in individuals who lived on Sumatra Island. The data also showed that the unmet dental care need of those who lived in Java Island decreased from year to year. This might be due to the availability of dentists that are more and more concentrated in Java Island, where the country's capital city lies. Therefore, dentists might not be as available in other areas. From this study, we could see that Susenas data is useful as a basis of policy recommendation for evaluating and regulating annual allocation of dentist based on dental care need of each area. Therefore, the geographic barriers to dental care might be reduced.

The utilization of dental care starts to increase from 2005 to 2007. These might be due to the implementation of the government's policy on Askeskin (insurance for the poor). Askeskin, which is heavily subsided by the government, started on January 2005. Before this policy was implemented, community participation in health insurance, which included primary dental care, reached only 26.9% [3]. Therefore, our data suggest that the implementation of Askeskin, which expended health insurance coverage including basic dental care, might reduced the Indonesians citizens' economic barriers to assessing dental care especially for the poor.

The results showed that dental health care requires more attention than it is currently receiving. Dental health prevention and promotion programs shall be encouraged to improve Indonesia's dental health. Government policy should highlight specific intervention programs that are feasible and sustainable to increase Indonesia's dental health through collaborative partnership, such as with dental professionals and local government. The results provided in this study could be used to identify groups that should be targeted specifically for dental health programs.

This study does, however, possess certain limitations. The first limitation arises from the use of the single item question about dental care needs. A “no” response is expected to mean that there was no dental problems. However, this same “no” response may also derive from different explanations such as, differences in pain sensitivity or because that their might be dental problems but were not painful, which did not actually signal to the host that the condition was a problem per se [11]. Despite the doubts expressed about single-item assessments, these assessments possess a number of distinct advantages. They are simple, clear, and relatively easy to use than complex questioners, especially in cases in which administration and clinical scoring are not feasible and brevity is essential [12]. Single-item indicators of dental health, like the one provided annually by the Susenas, provide benefits such as for monitoring and evaluating dental health needs and utilization, and for identifying determinants of dental health. 

The second limitation is that the measures of perceived dental care need were subjective, as they were based on the individual's perspective on dental illness. This could result in underreported objective need. “Need” could be conceptualized subjectively and objectively. Subjective need expresses the self-perceived need for treatment and varies from one individual to another, according to the sociocultural applicable. Objective need comes from the dentist's assessment, through identifying the signs of disease at an early stage, when no symptoms of oral disease have yet been noticed [13, 14]. Dental health perceptions may not only depend on one's sensitivity to signs of disease, but also may be influenced by an individual's knowledge of dental health [15]. The subjective dental assessment approach could be used to prioritize those really in need of dental care. Moreover, this approach provides a realistic estimation, since those who experience no perceived impact may not demand dental treatment [10, 16].

The study was limited to the variables included, because there was no availability to other sources of variables, which accounted as a nationally representative data for dental health. This might be a limitation of using Susenas, a national representative survey on social and economic status, which could not provide all the additional variables that might be needed for further dental care analysis. Despite of this, Susenas showed us its potential ability to be a proxy in describing Indonesias dental health in general. The reasons for using Susenas data are to obtain the large sample size. The Susenas data was invaluable because dental professionals may not be able to examine such a large population.

From these potential biases, Susenas still has weaknesses to measure dental care need of the Indonesian. Despite of the weaknesses, it is feasible and sustainable to be analyzed as a proxy of dental health in overall Indonesia. Moreover, in a time of limited resources, as is currently the case in Indonesia, it may be more important to identify those for whom health services will produce the most health gain. In this case, the use of subjective indicators as screening instruments, as provided by Susenas, looks much more promising. They provide a rapid and inexpensive way of determining who would benefit from a referral for professional attention. Therefore, Susenas is making annually evaluation of dental care in Indonesia possible. Objective needs are traditionally used to provide measures of dental health status for policy decisions. However, for a large country such as Indonesia, it is almost impossible to undertake this kind of survey annually. It has been suggested that perceived need plays a key role in whether people will seek dental care, and that a lack of perceptions regarding need constitutes an important barrier to the utilization of dental care services. Relying on clinical diagnosis alone, without integrating the psychosocial dimensions of dental health, would seriously overestimates the need for dental care [17]. Accordingly, objective measures of dental care need estimated by converting clinical measures alone would probably yield a result that is too high to be met in the Indonesian context, where the government's dental health care budget is inadequate to meet the entire dental care needs of the population. Although the disadvantages of employing Susenas, this study showed that Susenas was able to be a proxy that described Indonesians dental health care and was also able to dynamically assess the effect of government policies on dental health. In spite of that, further studies are needed to describe dental health care in Indonesia by analyzing both secondary and primary data for a more in-depth analysis.

## Figures and Tables

**Table 1:** Percent of respondents of the *Susenas* 2003–2007 by selected variables.

Variables	Year of survey										
	2003		2004		2005		2006		2007		Average
	(*N* = 895,427)		(*N *= 1,030,250)		(*N *= 1,052,091)		(*N *= 1,107,594)		(*N *= 1,167,019)		
	%	*N*	%	*N*	%	*N*	%	*N*	%	*N*	%
Perceived need for dental care											
No need	98.00	877,546	97.39	1,003,359	97.80	1,028,969	97.67	1,081,833	97.72	1,140,419	97.72
Need	2.00	17,881	2.61	26,891	2.20	23,122	2.33	25,761	2.28	26,600	2.28
Utilization of dental care											
Not used	99.34	889,483	99.09	1,020,913	99.40	1,045,742	99.35	1,100,392	99.16	1,157,212	99.27
Used	0.66	5,944	0.91	9,337	0.60	6,349	0.65	7,202	0.84	9,807	*0.74*

**Table 2:** Unmet dental care needs among Indonesian, *Susenas* 2003–2007.

Variables	2003		2004		2005		2006		2007		Average
	%	*N*	%	*N*	%	*N*	%	*N*	%	*N*	%
Had unmet dental care needs	66.76	11,937	65.28	17,554	72.54	16,773	72.04	18,559	63.13	16,793	72.04
Had dental visit	33.24	5,944	34.72	9,337	27.46	6,349	27.96	7,202	36.87	9,807	32.06

Total perceived need		17,881		26,891		23,122		25,761		26,600	

**Table 3:** Distribution of the *Susenas* respondents from 2003 to 2007 who had unmet dental care needs by age groups, gender, residence, macroregions, and health insurance entitlement.

Variables	2003 (*n* = 11, 937)	2004 (*n* = 17, 554)	2005 (*n* = 16, 773)	2006 (*n* = 18, 559)	2007 (*n* = 16, 793)
Age (years)	*P*-value < 0.001	*P*-value < 0.001	*P*-value < 0.001	*P*-value < 0.001	*P*-value < 0.001
<15	2,537 (21.3)	3,978 (22.7)	3,421 (20.4)	3,779 (20.4)	3,463 (20.6)
15–29	3,407 (28.5)	4,827 (27.5)	4,686 (27.9)	5,174 (27.9)	4,370 (26.0)
30–44	3,139 (26.3)	4,943 (28.2)	4,816 (28.7)	5,076 (27.4)	4,887 (29.0)
45–59	2,117 (17.7)	2,733 (15.6)	2,844 (17.0	3,282 (17.7)	2,946 (17.5)
60<	737 (6.2)	1,073 (6.1)	1,006 (6.0)	1,248 (6.7)	1,137 (6.8)
Gender	*P*-value > 0.001	*P*-value > 0.001	*P*-value > 0.001	*P*-value > 0.001	*P*-value > 0.001
Male	5,867 (49.2)	8,673 (49.4)	8,383 (50.0)	9,264 (49.9)	8,518 (50.7)
Female	6,070 (50.9)	8,881 (50.6)	8,390 (50.0)	9,295 (50.1)	8,275 (49.3)
Residence	*P*-value < 0.001	*P*-value < 0.001	*P*-value < 0.001	*P*-value < 0.001	*P*-value < 0.001
Urban	3,647 (30.6)	5,441 (31.0)	4,733 (28.2)	5,234 (28.2)	4,476 (26.7)
Rural	8,290 (69.5)	12,113 (69.0)	12,040 (71.8)	13,325 (71.8)	12,317 (73.4)
Macroregions	*P*-value < 0.001	*P*-value < 0.001	*P*-value < 0.001	*P*-value < 0.001	*P*-value < 0.001
Maluku Islands and West Papua	414 (3.5)	554 (3.2)	992 (5.9)	1,306 (7.0)	1,068 (6.4)
Lesser Sunda Islands	1,566 (13.1)	1,864 (10.6)	1,567 (9.3)	1,465 (7.9)	1,450 (8.6)
Kalimantan	1,305 (10.9)	1,988 (11.3)	2,415 (14.4)	2,108 (11.4)	1,991 (11.9)
Sulawesi	1,975 (16.6)	2,931 (16.7)	3,086 (18.4)	3,507 (18.9)	3,775 (22.5)
Java	3,558 (29.8)	4,498 (25.6)	3,684 (22.0)	4,085 (22.0)	3,021 (18.0)
Sumatra	3,119 (26.1)	5,719 (32.6)	5,029 (30.0)	6,088 (32.8)	5,448 (32.7)
Health insurance entitlement	*P*-value < 0.001	*P*-value < 0.001	*P*-value < 0.001	*P*-value < 0.001	*P*-value < 0.001
Other insurances	317 (2.7)	540 (3.1)	171 (1.0)	147 (0.8)	89 (0.5)
Private employees insurance	333 (2.8)	384 (2.2)	367 (2.2)	434 (2.3)	330 (2.0)
Government employees insurance	603 (5.1)	809 (4.6)	573 (3.4)	735 (4.0)	702 (4.2)
Insurances for the poor	1,752 (14.7)	3,252 (18.5)	2,174 (13.0)	4214 (22.7)	4,016 (23.9)
Uninsured	8,932 (74.8)	12,569 (71.6)	13,488 (80.4)	13,029 (70.2)	11,656 (69.4)

**Table 4:** Results of multivariate logistic regression analysis for unmet dental care needs with selected predictors from *Susenas* year 2003 to 2007.

Variables^∗^	2003 (*n* = 17,881)	2004 (*n *= 26,891)	2005 (*n *= 23,122)	2006 (*n *= 25,761)	2007 (*n* = 26,600)
	OR (95% CI)^§^	OR (95% CI)^§^	OR (95% CI)^§^	OR (95% CI)^§^	OR (95% CI)^§^
Age (years)					
<15	1.15 (1.00–1.31)	1.26 (1.13–1.66)	1.31 (1.16–1.47)	1.26 (1.12–1.41)	1.18 (1.06–1.31)
15–29	1.59 (1.40–1.81)	1.49 (1.34–1.66)	1.84 (1.63–2.08)	1.62 (1.45–1.81)	1.55 (1.39–1.72)
30–44	1.33 (1.17–1.52)	1.41 (1.27–1.56)	1.57 (1.40–1.77)	1.36 (1.22–1.52)	1.31 (1.19–1.45)
45–59	1.40 (1.22–1.61)	1.15 (1.03–1.29)	1.31 (1.16–1.48)	1.27 (1.14–1.43)	1.16 (1.04–1.29)
60<	**1** (reference)	**1** (reference)	**1** (reference)	**1** (reference)	**1** (reference)
Gender					
Male	**1** (reference)	**1** (reference)	**1** (reference)	**1** (reference)	**1** (reference)
Female	0.91 (0.86–0.97)	0.94 (0.89–0.99)	0.93 (0.88–0.98)	0.96 (0.91–1.01)	0.93 (0.89–0.98)
Residence					
Urban	**1** (reference)	**1** (reference)	**1** (reference)	**1** (reference)	**1** (reference)
Rural	1.41 (1.31–1.51)	1.17 (1.11–1.24)	1.23 (1.15–1.31)	1.17 (1.10–1.24)	1.12 (1.06–1.19)
Macroregions					
Maluku Islands and West Papua	**1** (reference)	**1** (reference)	**1** (reference)	**1** (reference)	**1** (reference)
Lesser Sunda Islands	0.93 (0.77–1.12)	0.71 (0.61–0.83)	0.55 (0.47–0.64)	0.52 (0.45–0.60)	0.52 (0.45–0.59)
Kalimantan	1.22 (1.00–1.48)	1.41 (1.20–1.66)	1.15 (0.98–1.34)	0.88 (0.76–1.01)	0.97 (0.85–1.11)
Sulawesi	1.31(1.09–1.58)	1.30 (1.11–1.51)	1.04 (0.90–1.20)	0.95 (0.84–1.09)	0.94 (0.83–1.07)
Java	1.01 (0.85–1.21)	0.99 (0.85–1.14)	0.72 (0.62–0.83)	0.76 (0.66–0.86)	0.66 (0.58–0.74)
Sumatra	1.24 (1.04–1.49)	1.38 (1.19–1.60)	1.10 (0.96–1.27)	0.91 (0.84–1.03)	0.75 (0.67–0.85)
Health insurance entitlement					
Other insurances	**1** (reference)	**1** (reference)	**1** (reference)	**1** (reference)	**1** (reference)
Private employees insurance	0.66 ( 0.52–0.84)	1.09 (0.89–1.32)	0.84 (0.62–1.13)	0.79 (0.57–1.08)	0.96 (0.68–1.34)
Government employees insurance	0.67 (0.54–0.84)	0.96 (0.82–1.12)	0.83 (0.63–1.11)	0.84 (0.62–1.14)	0.91 (0.66–1.26)
Insurances for the poor	0.97 (0.80–1.19)	1.59 (1.38–1.82)	1.08 (0.83–1.41)	1.01 (0.76–1.35)	1.33 (0.98–1.81)
Uninsured	1.37 (1.14–1.65)	1.97 (1.73–2.24)	1.58 (1.22–2.04)	1.45 (1.09–1.92)	1.75 (1.29–2.38)

^∗^ As explanatory variables, age, gender, residence, macroregions, and health insurance entitlement were input into the model as covariates by using a procedure for variable selection, in which all variables in a block are entered in a single step.

^§^ Adjusted odds ratios (ORs) and their 95% confidence intervals (CIs) were calculated using multiple logistic regression analysis. The met/unmet need for dental care was used as a response variable.
